# Efficacy and safety of topical ganciclovir in the management of cytomegalovirus (CMV)-related anterior uveitis

**DOI:** 10.1186/s12348-016-0078-z

**Published:** 2016-03-15

**Authors:** John X. H. Wong, Rupesh Agrawal, Elizabeth P. Y. Wong, Stephen C. Teoh

**Affiliations:** National Healthcare Group Eye Institute, Tan Tock Seng Hospital, Singapore, 308433 Singapore; Eagle Eye Centre, Singapore, Singapore

**Keywords:** Topical ganciclovir, CMV anterior uveitis, Recurrence of anterior uveitis

## Abstract

**Background:**

The aim of this study was to evaluate the efficacy and safety of topical ganciclovir 0.15 % gel in the management of patients with cytomegalovirus (CMV) anterior uveitis.

**Results:**

This was a retrospective cohort study of the disease course of 31 patients (33 eyes) with aqueous polymerase chain reaction (PCR) positive for CMV. Data from a total of 160 episodes of anterior uveitis flare for 20 years, dating from December 1992 to December 2012, was collected. All patients were treated with concomitant topical anti-inflammatory medication. The disease course of each eye was analysed before and after the use of topical ganciclovir 0.15 %. The mean age at initial presentation of anterior uveitis was 57.5 ± 12.6 years. Twenty-eight (90.3 %) patients were Chinese. Patients on topical ganciclovir gel had a statistically significant fewer episodes of uveitis flare per person year (median −0.88 episodes/person years, *p* = 0.029). The time-to-quiescence was not significantly affected by topical ganciclovir use (median −1.25 days, *p* = 0.610). In the survival analysis using the Cox regression model, the use of topical ganciclovir was associated with a lower risk of recurrence, but this was not statistically significant (hazard ratio = 0.857, 95 % CI 0.543–1.36, *p* = 0.511). The overall median time-to-recurrence was 290 days (95 % CI 113 to 274 days) and 164 days (125 to 404 days) (*p* = 0.492), with and without topical ganciclovir, respectively.

**Conclusions:**

Topical ganciclovir may be beneficial in reducing the frequency of recurrence in patients with CMV anterior uveitis, but it was not statistically associated with prolonging the time-to-recurrence. The time-to-quiescence was also not significantly affected by topical ganciclovir. Prospective studies with a larger number of patients would be required to verify our findings.

## Background

Cytomegalovirus (CMV) had been postulated as an etiological cause of hypertensive uveitis since the 1980s [1]. It was only in the past decade with the increased availability of polymerase chain reaction (PCR) that viruses have been confirmed as the aetiology of what was previously considered to be idiopathic anterior uveitis [2, 3].

The clinical manifestations of CMV infection of the anterior segment of the eye include anterior chamber inflammation, keratic precipitates (KPs), elevated intraocular pressure (IOP), and corneal endothelial cell damage [4]. The diagnosis of CMV anterior uveitis was by PCR of aqueous humour obtained via anterior chamber paracentesis.

In the treatment of patients with this disease entity, the discovery of this viral aetiology has lead to the possibility of including anti-viral medications to the treatment regime in addition to topical anti-inflammatory and topical anti-glaucoma medications Ganciclovir is a potent inhibitor of herpes viruses, including CMV. It is a nucleoside analogue that suppresses the replication of herpes viruses [5, 6] and functions as a virustatic agent. Ganciclovir has been used systemically, in the form of intravitreal injections and implants for CMV infections of the eye [7–9].

More recently, topical ganciclovir has been shown to be effective against herpetic keratitis [10]. Topical application of ganciclovir has also been shown to penetrate the corneal stroma and achieve therapeutic levels in the aqueous humour [11]. It has been used in the treatment of CMV anterior uveitis, but its effectiveness has been reported in only a limited number of studies [4, 9, 12].

We aim to evaluate the safety and efficacy of topical ganciclovir gel 0.15 % (Virgan, Laboratoires Théa, France) in the treatment of CMV anterior uveitis patients at a tertiary referral eye care centre in Singapore.

## Methods

We performed a retrospective chart review of patients with CMV anterior uveitis who were treated at our centre, from 1992 to 2012. This study was approved by the institutional review board (National Healthcare Group Domain Specific). We included eyes with clinical signs suggestive of CMV anterior uveitis including sentinel keratic precipitates, mild anterior uveitis, and high intraocular pressure. The eyes included were also positive for CMV on PCR from aqueous fluid and negative for herpes simplex virus (HSV), Varicella zoster virus (VZV), and *Toxoplasma gondii*. We excluded eyes with corneal involvement of the disease such as endotheliitis and corneal edema and eyes with CMV retinitis.

Topical ganciclovir 0.15 % gel was first made available at our centre in March 2010. We compared the disease course of each eye inflicted with CMV anterior uveitis before and after ganciclovir 0.15 % gel was added to the treatment regime. All the patients with positive PCR for CMV after March 2010 had treatment with topical ganciclovir 0.15 %.

Response to treatment or quiescence was defined as the reduction in anterior chamber (AC) inflammation to nil, with no episodes of flare-up of uveitis, which was defined as an increase in AC activity by one step. The primary outcome measures were time-to-quiescence, time-to-recurrence, and number of uveitis flare-ups per person year. Time-to-quiescence was defined as the duration from the presentation/recurrence of hypertensive uveitis to the time when zero cells were noted in the AC, as defined by the Standardization of Uveitis Nomenclature (SUN) Working Group [13]. Time-to-recurrence was defined as the duration between the start of one episode of hypertensive uveitis to the next episode. Crude incidence rates and recurrence rates were calculated, and comparisons were made for the disease course of each eye before and after the use of topical ganciclovir.

Patients were initially started on topical ganciclovir at an intensive frequency of 3 h (six times per day), tapered based on anterior chamber inflammation over the course of 3 months, and subsequently kept on long-term maintenance therapy (three to four times per day). All patients were concurrently treated with anti-inflammatory eye drops, in the form of topical steroids, and also topical anti-glaucoma medications as deemed necessary by the attending ophthalmologist. Topical non-steroidal anti-inflammatory drugs (NSAIDs) were not used in our cohort. None of the patients in this cohort had oral, intravitreal, or parenteral ganciclovir. The patients were reviewed weekly for the first month and then monthly thereafter, with more frequent reviews as necessary.

Data analyses were performed with Stata 11.0 (StataCorp LP, College Station, Texas, USA). Wilcoxon signed-rank test was used when comparing the differences within each eye while on and off topical ganciclovir. For survival analysis comparing the recurrences in eyes with and without topical ganciclovir treatment, a Cox regression model was constructed to analyse recurrences and to demonstrate efficacy of topical ganciclovir. Only the first eye which presented with CMV anterior uveitis in patients with bilateral disease was included in the survival analysis.

## Results

Data from 33 eyes of 31 patients were obtained. The mean age of these patients was 57.5 ± 12.6 years. The majority of our patients were male (74.2 %). The demographic characteristics of our patients are summarized in Table 1.

**Table 1:** Demographics of patients with CMV anterior uveitis

Mean age, years (SD)	57.5 (12.6)
Gender, *n* (%)	
Male	23 (74.2)
Female	8 (25.8)
Race, *n* (%)	
Chinese	28 (90.3)
Malay	2 (6.5)
Indian	0 (0.0)
Others	1 (3.2)

There were a total of 160 episodes of flare-ups of anterior uveitis in these patients. One hundred and seventeen episodes (36,440 days) were not treated with topical ganciclovir, while 43 episodes (11,750 days) were treated with topical ganciclovir. Patients were on maintenance treatment with topical ganciclovir for an average of 9.1 ± 2.5 months for each episode of uveitis flare. With topical ganciclovir use, there was a significant reduction in the median number of episodes of anterior uveitis recurrence per person year of −0.88 episodes per person year (episodes/PY) (*p* = 0.029). The median change of time-to-quiescence per episode of uveitis was −1.25 days (*p* = 0.610), and the median change of time-to-recurrence per episode of uveitis was −143.75 days (*p* = 0.285). These results are represented in Table 2.

**Table 2:** Comparison of median differences between Virgan use and no Virgan use

Virgan on vs Virgan off:	Median	IQR	*p* value
No. of episodes of uveitis recurrence per person year	−0.88	[−5.49–0.30]	0.029
Days-to-quiescence per episode of uveitis	−1.25	[−8.75–37.29]	0.610
Days-to-recurrence per episode of uveitis	−143.75	[−422.5–144.75]	0.285

**Fig. 1:**
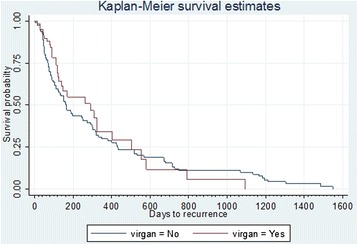
Kaplan-Meier survival estimates with and without topical ganciclovir

In the survival analysis using Cox regression model, topical ganciclovir was associated with a lower risk of recurrence but this was not statistically significant (hazard ratio = 0.857, 95 % CI 0.543–1.36, *p* = 0.511). The overall median time-to-recurrence, as represented in Fig. 1, was 290 days (95 % CI 113 to 274 days) with and 164 days (125 to 404 days) (*p* = 0.492) without topical ganciclovir use, respectively.

There were no significant side effects such as ocular discomfort from topical ganciclovir application or ocular surface toxicity noted in our patients.

Of the 33 eyes, 7 (21.2 %) developed raised IOP not controlled with maximum topical glaucoma medications and required glaucoma surgery. Four of the 7 eyes (57.14 %) had to undergo glaucoma surgery despite having been started on treatment with ganciclovir ointment. None of the patients developed corneal decompensation secondary to CMV anterior uveitis.

## Discussion

CMV anterior uveitis can result in significant visual morbidity due to its recurrent or chronic disease course [9]. The main complications include cataract formation, corneal decompensation, secondary open angle, and steroid induced glaucoma [14]. This disease entity affects the anterior segment of the eye only. Posterior segment involvement is not typically reported with CMV anterior uveitis. In a case series of five immunocompetent patients from Europe with positive CMV on anterior chamber paracentesis, none of them also had posterior segment involvement [15]. We have earlier reported clinical characteristics of 16 patients of CMV anterior uveitis in immunocompetent patients without any posterior segment involvement [16].

The role of topical therapy is pertinent as the disease is limited to the anterior segment without any posterior segment involvement. Chee and Jap reported that the use of topical ganciclovir in CMV anterior uveitis patients resulted in moderate response rates and lower recurrence rates as compared with systemic and implant ganciclovir [9]. Chronic prophylaxis was needed as hypertensive uveitis recurred after cessation of ganciclovir [8]. However, the duration of long-term therapy and its implications have not been reported in literature.

A recent report of 106 patients of CMV corneal endotheliitis by the Japanese corneal endotheliitis study group illustrated the clinical characteristics and treatment outcome for this type of viral uveitis [17]. The authors reported use of topical ganciclovir in 82 (75.2 %) eyes and systemic anti-CMV therapy in 74 (67.9 %) eyes. There were 52 (47.7 %) eyes treated with both systemic and topical anti-CMV drugs, and the results suggested that combination therapy is more beneficial. A significant number of patients (36 %) had recurrence of inflammation while on anti-CMV therapy. However, it was not analysed with duration of therapy [17].

The findings from our Cox regression model, in particular, the intra-eye comparison of the median number of episodes of anterior uveitis recurrence per person year with and without topical ganciclovir use and the overall median time-to-recurrence as represented in Fig. 1, suggested that the use of topical ganciclovir could reduce the number of episodes of uveitis recurrence (*p* = 0.029) but was not statistically associated with either the reduced risk of recurrence (*p* = 0.511) or prolonging the time-to-recurrence (*p* = 0.492). The time-to-quiescence was also not affected. These results contradict the findings of earlier reports and suggest that even though topical ganciclovir can reduce the number of recurrences of flare-up of uveitis in a PCR proven case of CMV anterior uveitis, it does not necessarily prolong the time-to-recurrence. This is the first ever reported Cox regression model for CMV anterior uveitis topical ganciclovir use. This model allowed us to study the recurrences over a period of time in person years, which is significant in the context of CMV anterior uveitis where there is no evidence in literature about long-term maintenance therapy or the safety and efficacy of topical ganciclovir as anti-CMV therapy.

The reduction in the median number of episodes of anterior uveitis recurrence of 0.88 episodes per person years may seem insignificant, but its potential cumulative effect and role in reducing glaucomatous damage or corneal decompensation in prolonging if not preventing the onset of sight threatening complications can be further explored in larger multicenter studies with expanded case definition of disease quiescence. However, patients have to be kept on maintenance therapy chronically. Repeated episodes of uveitis flare when patients were off topical ganciclovir may still result in progression of glaucomatous damage, which may necessitate glaucoma surgery.

Topical ganciclovir application is convenient and non-invasive as compared with intravitreal injections, implants, and systemic intravenous administration. There are no procedural risks involved. More importantly, the potential adverse effects of systemic ganciclovir and the ganciclovir implant limit their use on a long-term basis. There is no reported need for monitoring of full blood count and serum creatinine levels as compared to intravenous ganciclovir use.

This was a retrospective non-randomized study, and the study cohort was relatively small. The results need to be interpreted with caution and validated by larger trials. As data from this study was collected from 1992, the change in the type of topical steroids used over time could be a drawback of this study. Spersadexoline (dexamethasone 0.1 %, chloramphenicol 0.5 %, tetrahydrozoline HCl 0.025 %) was used before the availability of prednisolone acetate 1 % (Allergan, Irvine, USA).

## Conclusions

In conclusion, topical ganciclovir 0.15 % gel has a potential role in reducing episodes of recurrences in CMV anterior uveitis on long-term maintenance therapy. Reduction in number of recurrences per person year post treatment may be due to shorter follow-up period post treatment, and the heterogeneity in follow-up duration was identified as one of the limitations of this retrospective analysis. Hence, further prospective multicenter studies, with a larger number of patients required, to further explore the findings is needed to compare the safety and efficacy of topical gel with oral or intravitreal ganciclovir. Like herpetic keratouveitis, longitudinal cross-sectional studies are warranted for CMV anterior uveitis to determine the duration of long-term maintenance therapy.

## Abbreviations

AC: anterior chamber
CMV: cytomegalovirus
HSV: herpes simplex virus
IOP: intraocular pressure
KPs: keratic precipitates
NSAIDs: non-steroidal anti-inflammatory drugs
PCR: polymerase chain reaction
SUN: Standardization of Uveitis Nomenclature
VZV: Varicella zoster virus
